# Dementia Home Care Resources: How Are We Managing?

**DOI:** 10.1155/2012/590724

**Published:** 2011-10-29

**Authors:** Catherine Ward-Griffin, Jodi Hall, Ryan DeForge, Oona St-Amant, Carol McWilliam, Abram Oudshoorn, Dorothy Forbes, Marita Klosek

**Affiliations:** ^1^Arthur Labatt Family School of Nursing, Faculty of Health Sciences, University of Western Ontario (UWO), London, ON, Canada N6A 5C1; ^2^Health and Rehabilitation Sciences, Faculty of Health Science, UWO, ON, N6A 5C1, Canada; ^3^Faculty of Nursing, University of Alberta, AB, T6G 2R3, Canada; ^4^School of Health Studies, Faculty of Health Sciences, UWO, ON, N6A 5C1, Canada

## Abstract

With the number of people living with dementia expected to more than double within the next 25 years, the demand for dementia home care services will increase. In this critical ethnographic study, we drew upon interview and participant data with persons with dementia, family caregivers, in-home providers, and case managers in nine dementia care networks to examine the management of dementia home care resources. Three interrelated, dialectical themes were identified: (1) *finite formal care-inexhaustible familial care,* (2) *accessible resources rhetoric-Iinaccessible resources reality,* and (3) *diminishing care resources-increasing care needs*. The development of policies and practices that provide *available, accessible*, and *appropriate* resources, ensuring *equitable*, not necessarily equal, distribution of dementia care resources is required if we are to meet the goal of aging in place now and in the future.

## 1. Introduction

The number of people living with Alzheimer's disease or related dementia (ADRD) is expected to more than double within the next 25 years, affecting over 1.1 million Canadians and their families [1, 2]. Half of those with dementia live at home [3], and this proportion is increasing [4], especially amongst women [5]. Furthermore, families and friends are often the ones called upon to provide between 70%–90% of care [6] with adult children providing more care for their mothers than for their fathers. Clearly, the increasing number of people with ADRD will inevitably affect both formal and familial care for persons with ADRD who, on average, require care for approximately 8.5 years [7].

The challenges of providing home care have been well documented. Lack of support for family caregivers [8–11], lack of recognition and poor working conditions of home support workers [12, 13], early hospital-to-home discharge policy [14], and poor system coordination [15] are examples of the challenges faced by persons living with dementia and their familial and formal caregivers. Furthermore, limited resources to implement and sustain a home care infrastructure [16] and a shift of chronic care to community settings without the corresponding transfer of funds [17] compound the difficulties encountered. Although provincial government-funded home care agencies in Canada are mandated to assess and coordinate the health care needs of community-dwelling older adults with dementia, there is evidence that increasing caseloads among direct care providers [13] and case managers [18] have negatively impacted the quality of home care. 

In Ontario, 67% of formal home care has been provided by personal support workers and 27% by nurses, many of whom assume the role of case manager [19]. Case management, including assessment of client care needs and service allocation, coordination, monitoring, and evaluation based on assessed need, is a major strategy for home care management. Case management of home-based dementia care generally falls within one of three major models: (1) a brokerage model wherein case managers are charged with the responsibility of assessing the needs of clients, and based on these assessments, “decide, access, coordinate, monitor, and control amounts of timeframes of resources and services” [20, page 120]; (2) integrated service allocation and care managed by *teams of professional providers* who work directly with clients, sharing the responsibility and accountability for the management of both care and service allocation; (3) consumer-managed care, directed by the clients themselves, where clients select different mixes of services to achieve what they value [21]. All three models, however, typically rely on a range of human resources, which include family, friends, and neighbours (informal network), as well as professional and paraprofessional services (formal network). 

With the impending increase in the population of older persons in Canada, the availability of these human health resources to meet the growing demand for home care services is of rising concern to health care planners, policymakers, and other stakeholders [22, 23]. Furthermore, the need to both gain access to formal services and to sustain high levels of care may well exceed families' resources [24]. Consequently, as demands for home care escalate and the number of people living with dementia in the community increase, investigation into the management of dementia home care resources is critical if the goal of aging in place is to be realized. 

This article focuses on home-based dementia care from the perspective of persons with dementia, their family members, and home care providers, situating these experiences within a sociocultural context. In particular, we examined the relational experiences of clients, family caregivers, and providers involved in dementia home care, and the contextual factors that influence the formation and negotiation of those relationships, with the ultimate aim of identifying the necessary policy and practice changes to improve dementia home care. Four integral relational care processes emerged from our findings: *reifying care norms, managing care resources, making care decisions, *and *evaluating care practice.* This paper focuses on one relational care process, *managing care resources.*

## 2. Literture Review

Previous research on home support has identified that one of the challenges for home care workers is forming and maintaining relationships with clients and their family caregivers [25, 26]. Although the formal sector relies heavily on familial caregivers (families and friends) to provide the vast majority of care to persons with dementia, there has been little investigation of the relationship between the formal and informal home care networks. The few studies that have been conducted on home care [9, 27–30] have revealed an “uneasy alliance” and power struggles between the family caregivers and providers. Analysis of focus groups of 46 American home care clinicians revealed five inherent conflicts affecting family caregiver-clinician interactions: unrecognized family involvement; competing priorities and little time; lack of appropriate services to meet family needs; dual obligation of patient advocate and service gatekeeper; the reservation of social work services for difficult cases [9]. While collaboration is typically sought by both formal and familial caregivers, these individuals are situated in an emotionally charged “intermediate” domain, a contested area between the public world of paid care and the private world of family care [10, 11]. Thus, alliances between formal and familial care providers in home care are often formed under the guise of partnerships. 

Those studies that have explored home-based dementia care services have focused on the relationships between family caregivers and home care providers [31–33], family caregivers' expectations of providers [15], the use and satisfaction with home-based service for persons with dementia [34], and the relationship between formal and informal care systems [35]. In a mixed-method study, Forbes et al. [34] found that 39 family caregivers of persons with dementia identified availability and accessibility issues in seeking and using formal home care and community services in three provinces in Canada. The qualitative component of this study revealed challenges of service availability (e.g., lack of community based dementia care) and accessibility (e.g., lack of system coordination), however, our understanding into how the two care systems might “work together” in providing accessible and appropriate resources remains unclear. 

Although the relationship between the informal and formal care systems has been studied in a variety of settings, including home care [36–38], there are inconsistent findings about whether they substitute or complement one another. For instance, Zhu et al. [35] found that the utilization of formal home care and informal care was not influenced by the use of the other, and yet, there is empirical evidence that the two care systems are complementary [36, 37]. The theoretical basis and empirical support for both these approaches to understanding the link between formal and informal care systems are problematic [38]. First, the “partner relationship” between paid and unpaid care providers is espoused primarily by formal service providers and administrators as a way to limit financial expenditures of formal home services. In other words, the complementarity of the two systems is less compatible than is often assumed. Second, the analysis that guides most empirical studies on the relationship between formal and informal care systems is premised on the gendered assumption that the two are distinct entities, with family care rarely depicted as “care work”. This view not only simplifies the relationship between formal and informal caregiving, but it also fails to capture the specific commonalities and discerning differences between them. Thus, the differences between the two care systems with respect to power, status, authority, and resources are rarely considered in these studies. One exception, however, was Ward-Griffin and Marshall [38] who found home care nurses and family caregivers of older chronically ill elders living in Canada engaged in a bidirectional labour process of “work transfer”, one that depended on the “free” labour of family caregivers. Although this particular study sheds light on the complexity and interconnectedness of formal-informal care and points to the need to conceptualize eldercare as work, regardless of who does it, it did not focus on dementia home care. 

 There is also a need to examine the sociopolitical context of dementia home care because it permeates care systems at all levels [39]. For instance, a family member's “decision” to provide care to persons with dementia and to use formal care services depends on a number of sociocultural factors, such as the availability and cost of these services [40]. Family caregivers of persons with dementia also face a number of challenges in obtaining adequate, appropriate, consistent, flexible, and sufficient home care services [33, 34]. Since the conceptualization and availability of both informal and formal resources are profoundly shaped by the beliefs, values, practices, and policies within the current home care context, further investigation is warranted. 

In summary, there is limited research that examines the sociopolitical context of dementia home care. Given the increasing use of home care services, the projected decreasing availability of family caregivers and the increasing number of persons with dementia health care practitioners and policy makers would benefit from an increased understanding of the perceptions and experiences of persons with dementia, and their familial and formal care providers with regards to the management of home care resources.

## 3. Study Design

The purpose of this critical ethnography was to develop a better understanding of home-based dementia care within a sociocultural context, shedding light in particular on those sociocultural, taken-for-granted values, beliefs, and practices embedded in the dementia home care culture. 

### 3.1. Setting

As in other Canadian provinces, home care in Ontario entails the delivery of a wide range of health services to people of all ages who may be recovering from an illness, disabled or terminally ill. The central agency through which families access community care begins to manage a “case” by triaging it into one of five care domains: acute care, rehabilitation, maintenance, long stay supportive, and end-of-life care; seniors with dementia are usually classified into maintenance or long-stay supportive categories. Thereafter, care planning begins with a standardized assessment of the person with dementia's functional independence, couched within a goal-oriented model of client empowerment that aims to match services with assessments and with client preferences and strengths. As such, individuals receiving home care may be provided with medical, nursing, social, or therapeutic treatment or with assistance with essential activities of daily living. The fiscal constraints that characterize the province of Ontario's health care, however, preclude the provision of assistance with instrumental activities, such as housework, shopping, or meal preparation.

### 3.2. Recruitment

Case managers of the local Community Care Access Centre (CCAC) and other community collaborators served as key informants assisting with the identification and recruitment of potential procedures. All persons involved in the “caregiving network” of nine persons with dementia were requited for this study. To be included in the study, clients with Alzheimer's disease or related dementia had to speak and understand English, be at least 60 years of age or older, and have at least one family member or close friend providing regular care (minimum of 4 hours of direct or indirect care per week). Once the client and family caregiver(s) agreed to participate in the study, the home care worker(s) assigned to their care, for a minimum of three home visits, was invited to participate in the study by a member of the research team.

### 3.3. Sample

The total sample was comprised of nine networks, which included nine older adults with dementia, 25 family caregivers, 10 formal healthcare providers, and 7 CCAC case managers. The nine persons with dementia (PWD), four of whom were male, ranged in age from 75 to 91 (average 83.7 years). All but one were married, one was a widow, three had postsecondary education, one had only primary education, and the remaining PWD had secondary education. Standardized Mini Mental State Exam (SMMSE) scores ranged from 10/30 to 26/30, averaging 15.8 (*n* = 5; four scores were not obtained due to participants' relocation, refusal, or confusion). All PWD and their families were white and of Anglo-Saxon descent. Of the 25 family members who were spouses (*n* = 5,3 of whom were male), the average age was 78.8 years. Among the adult children (*n* = 20, 15 of whom were female), the average age was 50.1 years. All but three adult children worked full time. 

All nine networks were receiving formal home care support from personal support workers (PSWs). At least one PSW was recruited for each network (except network 9); for networks 3 and 8, two PSWs were recruited. In total, ten PSWs were recruited, two of whom were male. The average age of the PSWs was 52.8 years. Four worked full time, the others worked part time or on a casual basis. All seven home care case managers were female and of Anglo-Saxon decent. Their ages ranged from 45 to 57 (average: 50.9) years, and they had worked between 4 and 20 (average: 9.4) years as a case manager. Four of the managers coordinated caseloads that were primarily urban (i.e., within a large city in southern Ontario); the other three case managers provided services for those living in the surrounding counties (i.e., small towns and rural settings).

### 3.4. Data Collection

A total of 52 in-depth, semistructured interviews were conducted with clients, family caregivers, and PSWs. Each participant was interviewed two to threetimes over a period of 19 months (January 2007–July 2008). At the beginning of the initial interview, participants were asked to complete a brief demographic questionnaire, and for each person with dementia, an SMMSE score was obtained to describe the level of cognitive impairment. With the exception of the focus group interview with the case managers (which was conducted at the local CCAC office) and two interviews conducted by phone, all the interviews were conducted in the home of the client and/or family caregiver. After each interview, researchers dictated full field notes about their observations, perceptions, insights, nuances of communication, nonverbal expressions, caregiving behaviors, and interactions between and among all participants [41].

### 3.5. Data Analysis

Following the guidelines for data analysis of Lofland et al. [42], emerging initial codes were identified from the transcripts and the field notes. Memos were used as supplementary notes and background information to inform the analysis. After an iterative process of refining the initial codes, focused codes were applied to “clean” transcripts in an attempt to identify gaps or missing codes. The final analysis steps involved the development and refinement of the major themes, the identification of phrases that most accurately illustrated these themes, and diagramming, a process that facilitated an understanding of how the focused codes related to each other in order to conceptualize the larger picture [42].

## 4. Findings

Based on our analysis, three interrelated, dialectical themes were identified: (1) *finite formal care-inexhaustible familial care*, (2) *accessible resources rhetoric-inaccessible resources reality*, and (3) *diminishing care resources-increasing care needs. *Although the management of dementia home care resources is complex, study findings suggest that the lack of available, accessible, and appropriate formal care resources has ultimately contributed to the failure to provide optimal home-based dementia care.

### 4.1. Finite Formal Care—Inexhaustible Familial Care

Study findings revealed that the resource allocation by the formal sector depended heavily on families and friends to provide the bulk of care and assistance to persons with dementia, and only as a last resort, were formal care resources utilized to supplement this care. The notion of formal care resources as scarce commodities produced, by default, a view that familial care resources were abundant. Furthermore, the need for formal care assistance tended to be based on the assumption that family members were not only capable, but (ought to be) willing to provide care to members with dementia. Although all study participants addressed this “unspoken” reality that families provided the necessary care first and foremost, case managers were positioned to actively cultivate the availability of familial care resources. As one case manager explained:

I have a new demented person and the first thing that I did is to try to rally every family member… and gather as much family or friends that you can to start off with and focus on and get as much care in there as needed.

Care resources provided by the formal sector were viewed as supplementary “help” to the family, being made most readily available when the family member was viewed as “desperate” or in response to a crisis. In an extreme situation, one spousal caregiver explained how formal care resources were withheld until she secured the assistance of a physician:

And she [nurse] said, “Is he [person with dementia] in agony?” And I said, “Well, I don't know.” “Well when he gets in agony, call me back.” And I said, “I beg your pardon?” So I called the neurosurgeon... The gal on the desk answered it but she gave the phone to him, and he said, “What's going on?” and I said, “Thomas's catheter is plugged… and the [nurse] told me that I was to wait until he was in pure agony, and then call her back.” And he said, “I beg your pardon? Do you have her phone number?” And I said, “Yes I have.” “Well you give it to me. I'll get back to you.” Within 10 minutes he called back… and he said, “Sit back and relax…she's coming in.” But that kinda thing, you know? Who needs it? Nobody!

Furthermore, many family members reported feeling unheard when they expressed concerns or an inability to continue providing care. Another spousal caregiver stated that she only received “help” from the formal care system when she threatened to stop providing unpaid care:

My breathing has been terrible since I had my heart attack … and I've been begging for help … Nobody listened until now. And Jill [case manager] called me when I was trying to fix supper and I couldn't breathe. And she said, “Joan, you don't sound very good!” I said, “No …Sorry Jill. I'm ready to give it up…I can't do this anymore. This is just too hard to do.” [She responded] “Oh no! Don't do that. No, no don't do that!” So I received extra help… But it makes you feel you are on your knees, all the time begging for some help.

Despite the potential for “caregiver burnout”, case managers were required to distribute formal care resources within the current fiscal constraints of home care. This approach to dementia home care limited them in providing adequate support, even when familial care is exhausted. As one case manager reported:

Like that caregiver burnout, drop dead thing, the back-up plan for [the care of] these memory clients, there isn't one. So I mean I think that's how we can do it [alleviate caregiver burden]…four hours a week is what we're going to provide and we do whatever we can to make it work.

Using a supplementary approach to managing dementia care resources, however, did not alleviate caregiver burden in this study. Instead, the provision of care to persons with dementia over long periods of time exhausted the capacity of most families. Expectations of families to provide complex, continuous care without adequate formal care resources disproportionately burdens families exacting financial and emotional consequences. One spousal caregiver described the devastating and costly toll on family capacity and stability.

I've got a husband at home with dementia… I cannot leave him. [My doctor asked me] “Well don't you have any family?” [and] I said, “Well, my family disowned me because I used them too much.” And, it's true.

To complicate matters, the amount of formal home care available to persons with dementia and their families was often based on different interpretations of “hours available” and “need”, with opinions varying amongst family caregivers, in-home care providers, and case managers. A personal support worker responded to the question of increasing the hours of home support workers as a means to provide respite for a spousal caregiver,

That [maximum amount of hours] would be 15 hours in a week. And I don't know if … CCAC would allow it … It is hard to tell. It's because that would be the maximum hours … as far as I know, it's 60 hours in a month.

Ultimately, case managers were positioned to recommend the ‘appropriate' amount and frequency of formal home care support, depending on their assessment of the situation and the degree of latitude granted by their employer. Although most case managers conveyed that they followed a ‘standardized approach' in the allocation of available formal home care resources, others reported that this was sometimes negotiable, depending on the needs of the family. As one case manager explained:

I'll go out to a home and they'll say “Oh, I know she [another family caregiver] gets three afternoons a week to go out and play bingo.” …then I kind of have to say, “Tell me what you feel you need.” Like…I'm not going to walk in and tell you that you're going to get twelve hours, it's what you need to continue doing this. So again, there's no hard fast rule.

The provision of formal home care resources for persons with dementia and their family caregivers in a timely way was also another challenge identified. There were health and safety consequences to families when formal care resources were unavailable, especially during the evenings and/or weekends. A spousal caregiver recalled a situation in which she required the assistance of her landlord during the evening to clean up after a toileting accident:

I don't know how it happened, but the walls, the toilet, everything was covered [in feces]. And he [person with dementia] is trying to get down there, not telling me, trying to clean it up. And of course he ends up with it all over him, his hands. So then he's getting the taps. So disgusting (whispers)… It took [the land lord] an hour and a half to clean that bathroom, put my laundry in for me, and I said to him, “Boy this is beyond the call of duty…And I really appreciate it.” He said, “Don't worry about it” … But it's embarrassing, not for me so much, as it is for him… but I had no one else to call!

A daughter caregiver further explicated the context of home dementia care delivery, reflecting the belief that there was only a certain amount of care available:

I mean I know everybody is stretched to the limit…Even when you do request more care you are very fortunate if you get it, because there aren't as many PSWs and nurses out there, they can make a whole lot more money in the hospitals, so trying to get a lot more care [is not realistic]…we were very fortunate that we got what we did as far as I can tell.

Furthermore, when familial care resources were limited or became exhausted, the consequences to the person with dementia were potentially life threatening, as the following quote from a spousal caregiver depicted:

… She was in the hospital twice in two months …just because I couldn't take care of her. The first time she was severely constipated, I don't know!? She was only there for a day. And then the second time, she hadn't eaten … and [was] not drinking and she got dehydrated.

In addition to providing the vast majority of day-to-day care to the person with dementia, our findings indicated that family caregivers also assumed a great deal of responsibility for managing the care of people with dementia. One PSW spoke of the coordination role a daughter played in caring for her mother, noting how this was the exception, as there were many others who were not resourced as well:

But you know, even though her mom is living independently…I think Jane [daughter] does spend a lot of time monitoring, controlling, planning, setting up her mom's appointments, you probably heard about the appointments. She regularly goes to her dentist and her optometrist and the hearing specialist and now the dermatologist … Jane is very particular about her mom's health and taking her to appointments. (Pause). You see so many other cases where that just doesn't happen or where people have been parked in an apartment by themselves and say “Well mom manages best as she can” and they just don't manage.

Some people with dementia were also aware of how their families provided and managed their care. One woman remarked that her daughter coordinates her care which she finds helpful:

She gives a lot … just in her own way…I don't know how she remembers all those things said in that book... Well it's quite a bit to remember. She's gotta have everything just so!

In summary, the approach to care resource management was a supplemental model of care wherein formal home care resources were positioned as finite and precious commodities while family resources were viewed as “cost-effective” and inexhaustible. Many families, however, did not have the available resources, time or skills to assume the care management of people with dementia living at home, and yet, that was what was expected of them in view of the limited formal care resources. Moreover the management of care resources, formal or familial, was particularly challenging if the resources were inaccessible.

### 4.2. Accessible Community Resources: Reality or Rhetoric?

Although caregivers, providers, and case managers spoke about the importance of accessing a variety of community dementia care resources, many family members did not know which services existed or how to access them. Our findings also identified many barriers that prevented existing services from being accessible, such as long waiting lists (e.g., respite), rigid eligibility criteria (e.g., home care support), or cost of services (e.g., transportation, adult day care programs). 

Services were not only difficult to access in a timely manner, but were often transient or nonexistent. For instance, many participants spoke about the need for accessing appropriate respite services, both in-home and within day programs. The case managers, however, claimed that the actual provision of respite was challenging because there was never enough respite to meet the needs of families when they actually need it. Therefore, it was not uncommon for families to have to book six months or more ahead of the time the service was required. One son spoke about the lack of access to appropriate, timely resources for this father (person with dementia) at a time when his family needs were the greatest.

…I was there that summer and I do recall a lot of our hard times trying to get that extra help. Trying to increase the time for my father …. Like it would take a burden off and then she [mother] could do some of the stuff that needed to be done around the apartment… I can tell you if, there was ever a time where you need a lot of help, that was it ….I think that was a really bad example of how the system [didn't work]… I don't think it worked great at all.

Accessing care resources in a timely way is also compromised by systemic barriers, such as the hierarchal structure of the home care system, busy caseloads, and infrequent contact between home care providers. For instance, if personal support workers had any concerns or questions, they are instructed to contact their managers at their specific agency, who, in turn, contacted the CCAC case managers. For example, one PSW encountered difficulties in contacting his case manager to discuss a proposed increase in his hours due to deterioration in the health of the family caregiver.

I leave her [agency manager] notes if I have a concern, and sometimes I've even called her with things. At least I tried to get through. She's very hard to get through to…she never seems to answer her phone…

In another situation, the spousal caregiver felt it necessary to make the drastic decision to move to another apartment building, so her husband could continue to receive care from the same home support worker when their “catchment area” had been rezoned. As her daughter explained:

Bobby [PSW] seemed to be a real big help in our family and when his territory was, changed, my mother and father moved so that they could maintain support and not have to go to a new person. Because she just did not want to lose that support, and my dad seemed to respond to him.

There was also a discrepancy between what case managers *said *was accessible, and the care experiences of persons with dementia and their families as they tried to access home and community care services. Interestingly, case managers described how they assisted caregivers in navigating the system to access services, which was in direct contrast to family members' stories of the difficulties they encountered and the lack of support they received. As one case manager explained:

As a case manager it's my job to help with system navigation, to help them access [resources/services], tell them what's available, but to let the caregiver make decisions as to what they feel they need as they progress, and to support them through this journey.

And yet, as a daughter caregiver elucidated, bringing support into the home was neither a transparent process, nor, at times, a user-friendly activity:

…If the support can be easily brought into… the home then I think that is preferable than taking a person out of the home…I don't think there is any comparison … and when I say easy [to navigate the system], I also mean like just the process itself who—who to call, how to do it and not make it a challenge or a huge difficulty for the family or the caregiver.

When formal care services were provided in a standardized and regimented way, they were often described as inadequate and substandard. Older adults and their families struggled to acquire additional, essential resources to fill in the substantial gaps left by the formal system. Families who were without access to appropriate, timely home and community services were particularly vulnerable. As one daughter caregiver described:

You know that was an additional expense [house cleaning] and I couldn't pay for it …[So] now they come every 2nd week, so things like that, house cleaning which seems to be really basic care, are huge … help because it's just that much time and energy that she [spousal caregiver] doesn't have to spend on something you know, where she's probably already exhausted.

Those who could not afford to buy additional supports were also less likely to complain about substandard or inept in-home care for fear of not receiving any assistance. As one spousal caregiver explained:

You just aren't comfortable saying it's poor care because I didn't think I'd get anyone else…And I couldn't go with nothing…So it was, “hang in there”.

Thus, although the rhetoric of the system of home health care suggests that resources essential to quality home based dementia care are universally accessible to persons with dementia and their families, unfortunately, our findings illuminate that this assumption is more rhetoric than reality.

### 4.3. Diminishing Care Resources-Increasing Care Needs

The final theme reflects the temporal nature of care resources, whereby diminishing care resources occurred, while care needs are simultaneously increasing. The adequacy of resources was often assessed on what types of formal and familial care resources were available in the past, and if they met the current needs of the person with dementia, without adequate provision for future care. As a son caregiver clarified:

She [mother, caregiver of father] has Mark [PSW] here, and she's paying [a housekeeper] to come in and take care of the basic stuff. So, as long as those things remain and everything stays the same, then those would be the key components to make it work. If those disappeared, then I think it would…throw her out of whack, actually. I mean, I know that I can't keep coming in here every day.

The preceding quotation illustrates how formal care resources needed to be monitored on an ongoing basis because the demands of care as well as the familial resources often change over time with the progression of the disease. Spousal and adult child caregivers may become physically and emotionally depleted, and financial resources may run low. Therefore, given the cumulative effects of dementia care giving, the same level of resources and services may be no longer adequate or readily available. A daughter caregiver described this shift:

We, we've come to realize in the last year, that my dad's health has changed more significantly than it had previous to this last year and my mom's physical capability and mine…you know, we're not big strong people who can make sure he gets bathed properly and that kind of thing. We don't have all the appropriate facilities. It would be great if we had all the money in the world and we could build a big huge house to keep everybody with all of the sort of facilities and handicap services and everything. That would be great, but that's just not a reality.

Challenges in providing care are therefore exacerbated when there is a constant risk that the resources presently available will cease to be available or will become *inadequate* as dementia progresses. What may have been adequate at one point is no longer adequate at another. As one PSW explained:

I find they [family caregivers of persons with dementia] need more help than they're getting now whether it's PSW hours or hours with the nurse or some type of a counselor going in there assessing situations more often… More people could be kept out of institutions if they had a little bit more home help… more resources, more people watching, more people, some sort of a method where people are on top of the situation more.

There was also a sense of collective resignation amongst families, providers, and case managers that the current dementia home care system was the “best it can be.” One case manager described her sense of powerless:

I think when I first started out as a case manager, especially with dementia care patients, I really had to learn that you should let go and sometimes things will just be the way they are…you can only do so much. I call it “crash and burn”. If somebody is going to crash and burn, it's going to happen and *there's only so much you can do*.   That was a hard thing to let go of, because we like to help, you know, we like to fix, but some things, we can't fix.

These words reflected an implicit assumption that the formal care system is there to “help” families who do not or cannot provide the bulk of care for persons with dementia. This reinforces the notion that the care of older adults in the community is a family issue, instead of being viewed, at least in part, as a system failure that requires a different approach to dementia home care.

## 5. Discussion

Dementia home care was portrayed by persons with dementia, caregivers, providers and case managers in nine dementia care networks as three interrelated, dialectical themes: *finite formal care-inexhaustible familial care, accessible resources rhetoric-inaccessible resources reality*, and *diminishing care resources-increasing care needs. *The study findings suggest that unless familial and formal home care resources are reconceptualized and managed differently in the future, the needs of persons with dementia and their family caregivers will be drastically compromised. The study findings reflect the experiences of clients, family caregivers, and providers who were primarily white, Anglo-Saxon, and therefore, cannot be assumed to reflect the experiences of persons from varied ethnic backgrounds. In spite of this limitation, these findings provide several insights for future directions in home care practice, policy, and research. 

### 5.1. Relationships between Formal and Familial Care Systems

A home care system that depends so heavily on familial care should recognize families as indispensable partners in dementia home care, not resources to be exploited [30, 43]. Concerns that formal care services will drive out the unpaid family care must also finally be put to rest because we know from this study and from others [35, 44] that this is not the case. Indeed, study findings suggest that the abilities of families to sustain these high levels of care may well exceed their resources in the future [45]. As Levine [46] aptly argues, “the ongoing push toward a health care system that uses public resources sparingly and family caregivers liberally” is no longer viable. However, as long as the sociocultural assumption that family care resources are unlimited and exploitable remains intact, policymakers will not likely view support for caregivers as a wise and prudent decision [47]. Therefore, by reconceptualizing the relationship between formal and informal care systems to one that is collaborative rather than supplemental and potentially exploitive, we begin to open up possibilities to create a more equitable environment for dementia home care.

Study findings also revealed that the taken-for-granted assumption of finite formal care and inexhaustible family care is particularly difficult for families with limited financial and familial care resources. Their energy and capacity to provide care became depleted at the time when family caregivers needed them the most—as their relative's cognitive ability progressively declined. While there are several clinical assessment tools to measure caregiver burden [48], the family's “capacity” to provide and sustain long-term home care was rarely considered in our study. This finding suggests the need to change the current home care policy to one in which case managers are allocated the time and tools to carefully and routinely assess the family's capacity to provide dementia care over time, with the ultimate goal of delivering individualized, comprehensive formal care services to persons with dementia and their families, particularly in the later stages of the disease. 

Although the inequitable distribution between formal and familial care results in substantial costs to caregiving families [49], these financial expenditures were rarely acknowledged in this study, a finding that is consistent with previous investigations [47, 50]. As the purse holders or gate keepers of the system [38], the case managers focused on the costs of *formal care* services, which were carefully assessed and allocated according to the “medical needs” of the client. Furthermore, the family caregiver, not the case manager, tended to be the primary person who managed the care resources, but with limited or no authority to ensure optimal care. In light of the study findings, it is not surprising that caregiver burden is inherent in this supplementary care model that overuses familial care resources to the point of exhaustion. Unfortunately, improving formal care services in ways that may enhance the quality of life of caregivers and those they care for tends to receive low priority in the current policy culture. Despite the benefits of reducing family care burdens by providing available and accessible formal care [47], the expectation that families not only must, but also ought to cope with minimal if any formal care continues to exist in in a context of home care where the responsibility for care continues to shift from the state to individual families. 

 This expectation for family caregivers to deliver the bulk of dementia home care is not only shortsighted, but unaffordable. As dementia rates continue to rise, the costs of providing care to persons with dementia living in the community warrant increased attention [9]. Offering choice on how to manage their care may both lower the costs of home care and enhance client independence [51, 52]. Therefore, the current case management approach may potentially undermine both client and family involvement as well as position family caregivers in precarious financial and emotional situations. More research on the economics of caregiving is necessary, not only to fully understand the financial and social costs incurred by families, but also to identify what supports families need today and in the future. 

To ensure adequate provision of formal care resources, however, equal attention must be paid to the recruitment and retention of a strong home care workforce. The current shortage of home care workers is troubling [23, 26]. Research efforts to understand the work issues and working conditions of home support workers and nurses are critical in understanding human home care resources in the future. Furthermore, we need to better understand how to attract and retain these workers [12, 26, 46], as well as how to promote collaborative relationships between and among clients, families, home care workers, and managers if we hope to address this issue in the future. In addition, a change from home care policies and practices that contribute to the vulnerability of home care workers to ones that give them the recognition and remuneration they deserve is overdue [12]. Thus, the identification and implementation of necessary policy and practice changes can hopefully create a space for familial and formal caregivers, many of whom are women, to begin to develop and enjoy a meaningful, collaborative caregiving relationship. Ultimately, the joint efforts by persons with dementia, practitioners, family caregivers, and policy makers will lead to an improved and equitable relationship between formal and familial caregivers and the systems they represent.

### 5.2. Accessibility of Home and Community Resources

Similar to other studies [53], our findings illustrated that inequitable access to formal care resources has contributed to the strain that familial caregivers experience while trying to cope with the demands of providing care. A consistent theme in the research literature is that people who might benefit from respite care do not use these services or only in small amounts [54]. The utilization of certain services is due to many contributing factors, but amongst the most prevalent reasons are ones that were revealed in this study: family caregivers are not made aware that the services exist, and existing programs are inaccessible, inconvenient, or expensive [53]. This finding highlights the importance of families knowing the number of formal home care hours that are available to them, the need for a formal, targeted system of communication, and awareness/education programs for caregivers. Furthermore, formal care services based on symptoms and disability assessment are not always related to an individual's actual care needs. For instance, an individual with a moderate level of dementia may have fewer unmet needs because they were able to be met by their care environment, whereas a person receiving higher levels of assistance may have many of their needs left unmet because of low levels of personalized care [55]. There were also marked differences with respect to resources within and available to the networks in our study, yet they were treated as though they have the same access to resources. As opposed to adult children, spousal caregivers may not have the same resources such as health, information, and confidence, or families who live in rural areas [56]. This study finding is congruent with those of others who have raised concerns about equitable access to home care services [50]. Thus, flexible programs and services must be offered if the needs of all families are to be met, irrespective of their composition or where they reside.

Similar to Pratt et al. [57], we found that increasing access to services involves considering the wider social context of caregivers and their relationships with, among persons with dementia, other caregivers and professionals in order to more meaningfully understand issues of access. One model that takes social context into account is the integrated, continuing care model as proposed by Forbes and Neufeld [58]. This type of model is only likely to work, however, if it is sufficiently flexible to accommodate the divergent needs of persons with dementia and their caregivers in a heterogenous society. Rather than placing the onus on families to provide the vast majority of human resources, a preferred approach is to view the care of persons with dementia as care that involves the equitable, not equal, distribution of resources. Furthermore, integration of familial and formal care is desirable only if it involves a genuine partnership between those who provide care, and not just a blurring of their respective roles. According to Blustein [59], the family is a system of care whose values, attitudes, and practices distinguish it conceptually, ethically, and emotionally from other sorts of care relationships, and any “partnership” between the two systems which integrates the values of formal and family care should be one that recognizes and preserves these differences. In addition, there is a need to respect the differences, as well as the commonalities, between formal care and family care, otherwise no one is well served. Therefore, a new home care model is needed that not only includes persons living with dementia and their family caregivers as genuine partners in care, but also embraces diversity, flexibility, real choice, and supportive services, within the context of a national home care program [50, 54].

### 5.3. Organization and Delivery of Dementia Home Care Resources

Clearly, we need new ways to think about and manage dementia home care resources. At the very least, families must receive the support services they require to prevent their need for costly specialized services and premature institutionalization of the person with dementia or their caregivers [60]. Furthermore, if we hope to address the challenges of dementia home care in the future, it is important to reorganize the ways in which home health services are funded, organized, and delivered in Canada [9, 50]. Funding must be provided so that there are necessary resources to enable home care programs that meet the long-term needs of persons with dementia and their families. Just as other provinces, jurisdictions, or nations that count on the home care system to alleviate acute resource constraints must, the time has come to move beyond the current four percent-funding formula of the health care budget allocated to home care [61] and to adequately fund Canadian home care programs [54]. 

As in other neoliberal states where austerity measures reduce the resources available for social and health care, Canadians have witnessed in the last fifteen years the offloading of once public social programs to mixed economies of public, private, and for-profit welfare [62]. Major shifts in health care financing and home care reforms have led to fewer home care services at the same time that case managers and direct care providers have larger case loads of clients with more complex needs. Study findings have illuminated a common theme of competing priorities and little time, with case managers negotiating the competing roles of advocate and service gatekeeper. Similar to Aronson and Smith's study [62] of social service managers in southern Ontario, study findings illuminate the “quiet” resistance of case managers and how they struggled to respond to the shrinking formal care resources available to them. Very few case managers took on an active advocacy role in our study; however, they did not passively accept their situation in this restructured environment. Although the personal support workers and managers talked about their limited abilities to respond to the structural inequities experienced by the family caregivers, a number of them employed certain strategies to ‘get around' the perceived unjust practices and policies inherent in the system. Therefore, it is important in future research to explore the structural barriers that disable case managers and other home care workers in advocating for equitable home care practices that would enable aging in place in later life.

## 6. Conclusion

With the shrinking welfare state, the notion of optimal care has been replaced by discussions around whether services are available/unavailable, accessible/inaccessible, and adequate/inadequate; however, all three are interwoven such that without available and accessible services, services cannot be considered adequate. Home-based dementia services must, at the very least, provide care resources that are accessible *and* available in order to be considered adequate. As our findings illustrate, people who have the least amount of resources and the least amount of accessibility are the ones most struggling with inadequate care resources. Consistent with Jenga, a board game of balance, these networks are often teetering on the brink of collapse, and as long as they do not fall apart then the resources are perceived to be adequate. This current supplementary model of dementia home care is not only unjust, but it is also not sustainable in the future.

 Study findings suggest that we need to engage in critical dialogue and working toward policies and practices that will result in available and accessible resources to ensure optimal “aging in place” home-based dementia care. To meet this goal, we must first challenge the current assumption that formal care is finite and family care is inexhaustible. Home care practices and policies need to take into account the family's capacity to provide complex care over time. Second, the provision of available and accessible resources, including respite, programs, and home support workers, is essential to support families who provide this care [4, 20, 58]. Furthermore, families require, at a minimum, clear and honest information on how to access resources. Third, formal care providers need to actively advocate with caregivers for equitable distribution, not equal distribution of formal care resources. Finally, family caregivers, formal care providers, policy makers, and researchers need to share a common vision for home care resource management and collaborate in order to optimize the health of clients and families in home-based dementia care as they age in place now and in the future.

## References

[1] Alzheimers’ Society of Canada (2010). Rising tide: the impact of dementia on Canadian society.

[2] Smetanin P, Kobak P, Briante C (2006). Rising tide: the impact of dementia in Canada 2008 to 2038.

[4] Cranswick K, Thomas D Elder care and the complexities of social networks.

[5] Forbes DA, Jansen SL, Markle-Reid L (2008). Persons with dementia: use of health care. *Canadian Journal on Aging*.

[6] Cranswick K, Dosman D Eldercare: what we know today.

[7] Schreiner AS, Morimoto T, Asano H (2001). Death and dementia. *International Journal of Geriatric Psychiatry*.

[8] Armstrong P (2002). Health care privatization: women are paying the price. *Canadian Women’s Health Network*.

[9] Hokenstad A, Hart AY, Gould DA, Halper D, Levine C (2005). Closing the home care case: clinicians’ perspectives on family caregiving. *Home Health Care Management and Practice*.

[10] Ward-Griffin C (2002). Boundaries and connections between formal and informal caregivers. *Canadian Journal on Aging*.

[11] Ward-Griffin C (2001). Negotiating care of frail elders: relationships between community nurses and family caregivers. *Canadian Journal of Nursing Research*.

[12] Nugent LS (2007). Can’t they get anything better? Home support workers call for change. *Home Health Care Services Quarterly*.

[13] Sims-Gould J, Byrne K, Craven C, Martin-Matthews A, Keefe J (2010). Why I became a home support worker: recruitment in the home health sector. *Home Health Care Services Quarterly*.

[14] Bauer M, Fitzgerald L, Koch S Hospital discharge as experienced by family carers of people with dementia: a case for quality improvement.

[15] Jansen L, Forbes DA, Markle-Reid M (2009). Formal care providers’ perceptions of home- and community-based services: informing dementia care quality. *Home Health Care Services Quarterly*.

[16] Health Council of Canada (2008). *An Update on Primary Health Care and Home Care Renewal in Canada*.

[17] Rajnovich B, Keefe J, Fast J Supporting caregivers of dependent adults in the 21st century.

[18] Dalby DM, Hirdes JP (2008). The relationship between agency characteristics and quality of home care. *Home Health Care Services Quarterly*.

[19] Ontario Home Care Association Ontario Home Care System in 2008: A Growing History of Quality and Excellence. http://www.homecareontario.ca/public/docs/news/2008/June/Ontario-Home-Care-Overview.pdf.

[20] McWilliam CL, Ward-Griffin C (2006). Implementing organizational change in health and social services. *Journal of Organizational Change Management*.

[21] McWilliam CL, Ward-Griffin C, Sweetland D, Sutherland C, O’Halloran L (2001). The experience of empowerment in in-home services delivery. *Home Health Care Services Quarterly*.

[22] Carrière Y, Keefe J, Légaré J, Lin X, Rowe G (2007). Population aging and immediate family composition: implications for future home care services. *Genus*.

[23] Milden B, Nelson S, Doran D (2011). Home care nursing workload: the what and why. *Mapping the Field: Nursing Scholarship in Health Human Resources*.

[24] Keating N, Dosman D (2009). Social capital and the care networks of frail seniors. *Canadian Review of Sociology*.

[25] Martin-Matthews A, Sims-Gould J (2008). Employers, home support workers and elderly clients: identifying key issues in delivery and receipt of home support. *Healthcare Quarterly*.

[26] Sims-Gould J, Martin-Matthews A (2010). Strategies used by home support workers in the delivery of care to elderly clients. *Canadian Journal on Aging*.

[27] Benzein E, Johansson B, Saveman BI (2004). Families in home care—a resource or a burden? District nurses’ beliefs. *Journal of Clinical Nursing*.

[28] Levine C (2004). *Always on Call: When Illness turns Families into Caregivers*.

[29] Stajduhar KI, Funk LM, Roberts D (2011). Articulating the role of relationships in access to home care nursing at the end of life. *Qualitative Health Research*.

[30] Ward-Griffin C, McKeever P (2000). Relationships between nurses and family caregivers: partners in care?. *Advances in Nursing Science*.

[31] Callahan CM, Boustani MA, Unverzagt FW (2006). Effectiveness of collaborative care for older adults with Alzheimer disease in primary care: a randomized controlled trial. *Journal of the American Medical Association*.

[32] Heinrich M, Neufeld A, Harrison MJ (2003). Seeking support: caregiver strategies for interacting with health personnel. *Canadian Journal of Nursing Research*.

[33] Neufeld A, Harrison MJ, Hughes K, Stewart M (2007). Non-supportive interactions in the experience of women family caregivers. *Health and Social Care in the Community*.

[34] Forbes DA, Markle-Reid M, Hawranik P (2008). Availability and acceptability of Canadian home and community-based services: perspectives of family caregivers of persons with dementia. *Home Health Care Services Quarterly*.

[35] Zhu C, Torgan R, Scarmeas N (2008). Home health and informal care utilization and costs over time in Alzheimer’s disease. *Home Health Care Services Quarterly*.

[36] Chappell N, Blandford A (1991). Informal and formal care: exploring the complementarity. *Ageing and Society*.

[37] Denton M (1997). The linkages between informal and formal care of the elderly. *Canadian Journal on Aging*.

[38] Ward-Griffin C, Marshall VW (2003). Reconceptualizing the relationship between “public” and “private” eldercare. *Journal of Aging Studies*.

[39] Szinovacz ME, Davey A (2007). *Caregiving Contexts: Cultural, Familial, and Societal Implications*.

[40] St-Amant O, Ward-Griffin C, DeForge R Making care decisions in home-based dementia care: why context matters.

[41] Morse JM, Field PA (1995). *Qualitative Research Methods for Health Professionals*.

[42] Lofland J, Snow DA, Anderson L, Lofland J (2006). *Analyzing Social Settings: A Guide to Qualitative Observation and Analysis*.

[43] Guberman N, Lavoie JP, Pepin J, Lauzon S, Montejo ME (2006). Formal service practitioners’ views of family caregivers’ responsibilities and difficulties. *Canadian Journal on Aging*.

[44] Penning MJ (2002). Hydra revisited: substituting formal for self- and informal in-home care among older adults with disabilities. *Gerontologist*.

[45] Keating N, Fast J, Frederick J, Cranswick K, Perrier C (1999). *Eldercare in Canada: Context, Content and Consequences*.

[46] Levine C, Murray T, Levine C, Murray T (2008). Building on common ground. *The Cutures of Caregiving. Conflict and Common Ground Among Families, Health Professionals and Policy Makers*.

[47] Feder J, Levine C, Levine C, Murray T (2008). Explaining the paradox of long-term care policy. An example of dissonant cultures. *The Cutures of Caregiving. Conflict and Common Ground Among Families, Health Professionals and Policy Makers*.

[48] Keefe J, Guberman N, Fancey P, Barylak L, Nahmiash D (2008). Caregivers’ aspirations, realities, and expectations: the CARE tool. *Journal of Applied Gerontology*.

[49] Fast JE, Williamson DL, Keating NC (1999). The hidden costs of informal elder care. *Journal of Family and Economic Issues*.

[50] Coyte PC, McKeever P (2001). Home care in canada: passing the buck. *Canadian Journal of Nursing Research*.

[51] Chapman SA, Keating N, Eales J (2003). Client-centred, community-based care for frail seniors. *Health and Social Care in the Community*.

[52] McWilliam CL, Stewart M, Vingilis E (2007). Can we afford consumers choice in home care?. *Care Management Journals*.

[53] Zarit SH, Gaugler JE, Jarrott SE (1999). Useful services for families: research findings and directions. *International Journal of Geriatric Psychiatry*.

[54] Forbes DA, Jansen SL, Markle-Reid M (2008). Gender differences in use and availability of home and community-based services for people with dementia. *Canadian Journal of Nursing Research*.

[55] Hancock GA, Reynolds T, Woods B, Thornicroft G, Orrell M (2003). The needs of older people with mental health problems according to the user, the carer, and the staff. *International Journal of Geriatric Psychiatry*.

[56] Forbes DA, Ward-Griffin C, Kloseck M My world gets smaller and smaller with nothing to look forward to: Dimensions of social inclusion and exclusion among rural dementia care networks.

[57] Pratt R, Clare L, Kirchner V (2006). ’It’s like a revolving door syndrome’: professional perspectives on models of access to services for people with early-stage dementia. *Aging and Mental Health*.

[58] Forbes DA, Neufeld A (2008). Looming dementia care crisis: Canada needs an integrated model of continuing care now!. *Canadian Journal of Nursing Research*.

[59] Blustein J, Levine C, Murray TH (2004). Integrating medicine and the family: toward a coherent ethic of care. *The Cultures of Caregiving: Conflict and Common Ground Among Families, Health Professionals and Policy Makers*.

[60] Cohen M, McLaren A, Sharman Z (2006). *From Support to Isolation: The High Cost of BC’s Declining Home Support Services*.

[61] Scarrow J (2007). Bringing health care home. *Registered Nurse Journal*.

[62] Aronson J, Smith K (2011). Identity work and critical social service management: balancing on a tightrope?. *British Journal of Social Work*.

